# Changes in the prevalence of obesity and hypertension and demographic risk factor profiles in China over 10 years: two national cross-sectional surveys

**DOI:** 10.1016/j.lanwpc.2021.100227

**Published:** 2021-07-31

**Authors:** Yongze Li, Di Teng, Xiaoguang Shi, Xiaochun Teng, Weiping Teng, Zhongyan Shan, Yaxin Lai, for the China National Diabetes and Metabolic Disorders Study Group and the Thyroid Disorders, Iodine Status and Diabetes Epidemiological Survey Group

**Affiliations:** Department of Endocrinology and Metabolism and the Institute of Endocrinology, First Hospital of China Medical University, Shenyang, 110001, Liaoning, China

**Keywords:** Obesity, Hypertension, Prevalence, China

## Abstract

**Background:**

Previous studies have shown increases in the prevalence of obesity and hypertension, but nationally representative data on recent changes in prevalence adjusted for population structure changes are lacking. Two nationwide surveys were conducted in 2007 and 2017 to assess the prevalence changes of these conditions in China.

**Methods:**

A multistage stratified random sampling method was used to obtain a nationally representative sample of adults aged 20 years and older in mainland China in 2007 and 2017. Temporal changes in the prevalence of hypertension and obesity were investigated. Changes in blood pressure, body mass index (BMI) and waist circumference were also assessed. Logistic regression models were constructed to assess the changes in prevalence over time.

**Findings:**

The weighted prevalence of hypertension (25.7% vs. 31.5%, *P*=0.04), high-normal blood pressure (11.7% vs. 14.3%, *P*<0.0001), general obesity (31.9% vs. 37.2%, *P*=0.008), and central obesity (25.9% vs. 35.4%, *P*=0.0002) was significantly higher in 2017 (n=72824) than in 2007 (n=45956) in the overall population. No significant changes in the prevalence of overweight and grade 1 or grade 2 hypertension were observed in the overall population, but a significantly higher prevalence was observed among participants aged 20-29 years for grade 1 hypertension (*P*=0.002) and among participants aged 70 years and older for grade 2 hypertension (*P*=0.046) in 2017.

**Interpretation:**

Compared with 2007, the prevalence of hypertension and obesity was significantly higher among adults in mainland China after adjusting for demographic confounding factors in 2017. More targeted interventions and prevention strategies are needed to offset the increasing risk of cardiovascular disease due to increases in the prevalence of hypertension and obesity.

**Funding:**

The Clinical Research Fund of the Chinese Medical Association (Grant No. 15010010589), the National Natural Science Foundation of China (Grant No. 82000753), and the Chinese Medical Association Foundation and Chinese Diabetes Society (Grant No. 07020470055)

**Research in Context**

Evidence Before this Study

A growing body of literature has documented increasing changes in the prevalence of obesity and hypertension among adults in China. However, the majority of these studies were limited to certain age groups or regions or involved nonrepresentative sampling. Dynamic changes in population structure, economic development, education levels, and lifestyles should be taken into consideration when assessing the changes in prevalence over time. In addition, the change in the prevalence of high-normal blood pressure in mainland China is unknown.

Added Value of this Study

Two large-sample nationally representative surveys indicated that the prevalence of general obesity, central obesity, hypertension, and high-normal blood pressure was higher in 2017 among Chinese people aged 20 years or older than in 2007.

The higher prevalence of hypertension and obesity shifted from urban to rural populations over the course of a decade.

Body mass index, waist circumference, and systolic blood pressure have increased slightly, with relatively larger increases in systolic blood pressure in men, rural residents, and young adults.

Implications of All the Available Evidence

This study revealed increasing changes in the prevalence of obesity and hypertension in Chinese adults, as well as the prevalence of high-normal blood pressure, indicating a substantial future burden of cardiovascular disease in China.

The changes in the populations most commonly affected by high-normal blood pressure suggest that increased attention should be given to men, young adults, and rural residents.

More targeted interventions and prevention strategies are needed to offset the increasing risk of cardiovascular disease due to increases in the prevalence of hypertension and obesity.

Alt-text: Unlabelled box

## 1 Introduction

Obesity and hypertension, which are two major risk factors for noncommunicable diseases (NCDs), contribute to global health and economic burdens.[1,2] The prevalence of obesity has increased worldwide in the past 50 years, and it is often referred to as an epidemic. Obesity represents a major health challenge because it substantially increases the risk of diseases such as type 2 diabetes mellitus, fatty liver disease, and several cancers, thereby contributing to a decline in both quality of life and life expectancy.[1] Moreover, overweight and obesity are associated with hypertension, and hypertension is considered to be the leading cause of cardiovascular disease and premature death worldwide.[3] Over the past few decades, obesity and hypertension have increased rapidly in Asian countries due to the westernization of lifestyles.[4], [5], [6], [7] In India, more than 135 million individuals are affected by obesity.[4] As a developing country, Vietnam is also facing several environmental and health problems, including hypertension and obesity.[5,6] Even in Japan, a country with a highly developed economy, hypertension is highly prevalent, affecting up to 60% of men and 45% of women.[7]

With the acceleration of China's economic development and urbanization, the ageing of the population and the ongoing epidemic of obesity, hypertension has become a major public health problem affecting Chinese residents.[8] Data from the China National Nutrition Surveys in 2015 indicate that the prevalence of obesity among adults in China was 16.4%.[9] The previous national survey of hypertension in China, conducted in 2017, found a hypertension prevalence of 44.7%.[10] In 2019, NCDs accounted for approximately 95% of all deaths and 90% of all disability-adjusted life years lost in China, which increased from approximately 80% and 60% in 1990, respectively.[11] The increase in obesity and hypertension is expected to continue to affect the future burden of NCDs.[12] A growing body of literature has documented increasing trends in the prevalence of obesity and hypertension among adults in China.[12], [13], [14] However, the majority of these studies were limited to certain age groups or regions or involved nonrepresentative sampling. In addition, dynamic changes in population structure, economic development, education levels, and lifestyles should be taken into consideration when assessing the changes in prevalence over time.

In June 2020, the International Society of Hypertension (ISH) published new guidelines for the management of patients with arterial hypertension.[15] Compared with the previous guidelines, the 2020 ISH guidelines have a simplified definition of the blood pressure categories, making classification and risk stratification of people with hypertension more feasible for clinicians.[16] Awareness of the risks associated with high-normal blood pressure needs to be promoted so individuals with this condition can delay or prevent incident hypertension through the early adoption of healthy lifestyle interventions that lower blood pressure levels and reduce the risk of cardiovascular disease.[15] However, the changes in the prevalence of high-normal blood pressure in mainland China are unknown.

To obtain a more accurate and comprehensive understanding of the changes in obesity and hypertension in mainland China over the decade between 2007 and 2017, this analysis presents nationally representative data from two population-based cross-sectional surveys. In addition, we determined the temporal changes in the prevalence of different categories of hypertension and obesity both in the overall population and within subgroups defined by sociodemographic and behavioural characteristics after adjusting for demographic confounding factors.

## 2 Methods

### 2.1 Study Population and Survey Design

The first national cross-sectional study (China National Diabetes and Metabolic Disorders Study) was carried out in 2007-2008 to evaluate the status of major metabolic risk factors, including blood glucose, blood pressure, and blood lipids, within the adult population of mainland China. Details of the study design are presented elsewhere.[17] In brief, a multistage stratified random sampling method was used to select a nationally representative sample of the general population aged 20 years or older in China (Supplementary Figure 1). An additional cross-sectional survey (Thyroid Disorders, Iodine Status and Diabetes Epidemiological Survey) was carried out in 2015-2017. We previously described the study design in detail, and a detailed flowchart of the study design can also be found in Supplementary Figure 1.[18] Briefly, the same multistage stratified random sampling method was applied in urban and rural locations to obtain nationally representative samples (Supplementary Figure 1). The inclusion criteria for this study were as follows: age 20 years or older, having lived in the selected community for at least five years, and not pregnant. Ultimately, 45956 participants in 2007 and 72824 participants in 2017 were eligible for inclusion in the analysis after the exclusion of those with missing information on sex, age, body mass index (BMI), waist circumference, systolic blood pressure (SBP), and diastolic blood pressure (DBP) (Supplementary Figure 1). The numbers of participants with missing information were 283 (0.6%) in 2007 and 524 (0.6%) in 2017. These missing data were not associated with either the specific value that was supposed to be obtained or the set of observed responses. The analysis thus remains unbiased. Power may be slightly lost in the design, but the estimated parameters are not biased due to missing data.[19] The research protocols were approved by the medical ethics committees of China Medical University and China–Japan Friendship Hospital. All the participants provided written informed consent after receiving a thorough explanation of the research procedures.

### 2.2 Measurements

For each participant, a trained interviewer used a detailed questionnaire to collect information about demographic variables, behavioural factors, and personal medical history. Current smoking was defined as having smoked at least 100 cigarettes in one's life and currently smoking cigarettes. An identical protocol was used to measure body weight, height, and waist circumference in 2007 and 2017. Body weight and height were measured according to the 3rd edition of Cardiovascular Survey Methods from the World Health Organization (WHO).[20] BMI was calculated by dividing the body weight in kg by the square of the height in metres. Waist circumference was measured in upright participants midway between the lower edge of the costal arch and the upper edge of the iliac crest.[20]

In the first study, blood pressure was measured using a standardized calibrated mercury sphygmomanometer (regular adult, large, or thigh) in the seated position after five minutes of rest.[20] Two consecutive readings were taken on the nondominant arm. In the second study, blood pressure was measured by a validated electronic blood pressure monitor (Omron HEM-7430, Omron Corporation) on the nondominant arm with the participant in a seated position after five minutes of rest.[20] Two consecutive measurements were taken with a 10-minute interval between measurements. The mean of the two consecutive measures was used in both studies for analysis.

### 2.3 Definitions of Obesity and Hypertension

According to the International Diabetes Federation diagnostic criteria, we defined central obesity as a waist circumference of 90 cm or greater for men and 80 cm or greater for women.[21] According to the Asian-specific cut-off points, overweight was defined as a BMI from 23 kg/m^2^ to less than 25 kg/m^2^, and general obesity was defined as a BMI of 25 kg/m^2^ or greater for both men and women.[22] According to the 2020 ISH guidelines, hypertension was defined as an SBP of 140 mmHg or greater, a DBP of 90 mmHg or greater, or the self-reported use of antihypertensive medication within the previous two weeks.[15] Normal BP was defined as an SBP less than 130 mmHg, a DBP less than 85 mmHg and no use of antihypertensive medicines.[15] High-normal BP was defined as an SBP from 130-139 mmHg, a DBP from 85-89 mmHg, and no use of antihypertensive medicines.[15] Grade 1 hypertension was defined as an SBP from 140-159 mmHg and/or a DBP from 90-99 mmHg.[15] Grade 2 hypertension was defined as an SBP of 160 mmHg or greater and/or a DBP of 100 mmHg or greater.[15]

### 2.4 Statistical Analysis

An identical statistical plan was used to account for the complex sampling design of the two studies; we used SUDAAN software (Research Triangle Institute) to obtain prevalence estimates and standard errors according to the Taylor linearization method.[23] The Taylor series (linearization) method is the most commonly used method to estimate the covariance matrix of the regression coefficients for complex survey data.[23] Estimates were weighted to reflect the age, sex, and urban-rural distributions of the geographic regions of the adults living in China. Weighting coefficients were derived from the Chinese population census data, and the sampling scheme of the two surveys was used to obtain a national estimate. Briefly, the weighting coefficient was the inverse of the adjusted probability of obtaining the data for the respondent; each individual case in the analysis was assigned a specific coefficient (individual weight), by which the value was multiplied to represent the actual population with the same characteristics of sex, age, province, and location. Standard errors were estimated by Taylor series linearization. To counteract the effect of the changes in population structure from 2007 to 2017, age- and sex-specific adjustments were performed using direct standardization, with the standard being all adults across the entire period; the age- and sex-specific standardized coefficients were based on the 2010 Chinese population census data. Categorical data are presented as percentages and 95% confidence intervals (CIs) and were analysed by a *χ*2 test or Fisher's exact test, as appropriate. Continuous data are described as the means and 95% CIs and were analysed with *t* tests. Logistic regression models were used to examine the changes in the prevalence of obesity and hypertension between 2007 and 2017. Linear regression models were used to estimate the changes in mean SBP, DBP, BMI, and waist circumference between 2007 and 2017. To further test the stability of the results, two sets of sensitivity analyses for odds ratios (ORs) were undertaken. First, three models with progressively increased adjustment of risk factors among all participants were applied. Second, considering that the prevalence of obesity and hypertension differs according to demographic background, we stratified participants according to subgroups for analysis. Statistical significance was defined by a 2-sided *P* value <0.05. All the statistical analyses were conducted using SAS, version 9.3 (SAS Institute Inc, Cary, NC) and SUDAAN, version 10.0 (Research Triangle Institute).

### 2.5 Role of the Funding Source

The funders had no role in the execution of this study or the interpretation of the results.

## 3 Results

### 3.1 Characteristics of the Study Participants

Table 1 presents the characteristics of the respondents in each survey. Significant differences were observed in the mean age, sex, income level, education level, BMI, waist circumference, and SBP levels between 2007 and 2017. Compared with 2007, the mean age was younger (44.8 years vs. 43.8 years, *P*=0.02) in 2017; the proportion of men was higher (49.4% vs. 50.2%, *P*=0.0001) in 2017. Higher income levels, education levels, BMI values, waist circumferences, and SBP levels were seen in 2017 (*P*<0.05 for all).

**Table 1:** Sample characteristics (weighted) by survey wave. Values are percentages (95% CI) unless stated otherwise.

Characteristics	2007	2017	*P* for difference
No. of participants	45956	72824	
Mean age at survey (standard error), years	44.8 (0.14)	43.8 (0.42)	0.02
Sex			
Men	49.4 (49.3 to 49.5)	50.2 (49.9 to 50.5)	
Women	50.6 (50.5 to 50.7)	49.8 (49.5 to 50.2)	0.0001
Urbanization			
Urban	45.8 (18.6 to 75.8)	52.1 (31.7 to 71.8)	
Rural	54.2 (24.3 to 81.4)	47.9 (28.2 to 68.3)	0.75
Ethnicity			
Han	86.4 (69.2 to 94.7)	95.6 (93.9 to 96.8)	
Non-Han	13.6 (5.3 to 30.8)	4.4 (32 to 6.1)	0.14
Income per year			
≤30000 yuan	82.8 (77.7 to 87.0)	44.7 (38.0 to 51.5)	
>30000 yuan	17.2 (13.0 to 22.4)	55.3 (48.5 to 62.0)	<0.0001
Education			
Less than college	79.6 (72.5 to 85.2)	66.2 (59.1 to 72.7)	
College and above	20.4 (14.8 to 27.5)	33.8 (27.3 to 40.9)	0.006
Current cigarette smoker	26.1 (23.5 to 28.8)	26.7 (25.4 to 27.9)	0.69
Physical examination, mean (standard error)			
Mean body mass index, kg/m^2^	23.7 (0.17)	24.1 (0.06)	0.04
Mean waist circumference, cm	80.7 (0.58)	83.5 (0.37)	0.0002
Mean systolic blood pressure, mm Hg	121.7 (0.79)	126.6 (0.67)	<0.0001
Mean diastolic blood pressure, mm Hg	77.5 (0.61)	78.6 (0.57)	0.23

### 3.2 Changes in the Prevalence of Obesity

**Figure 1:**
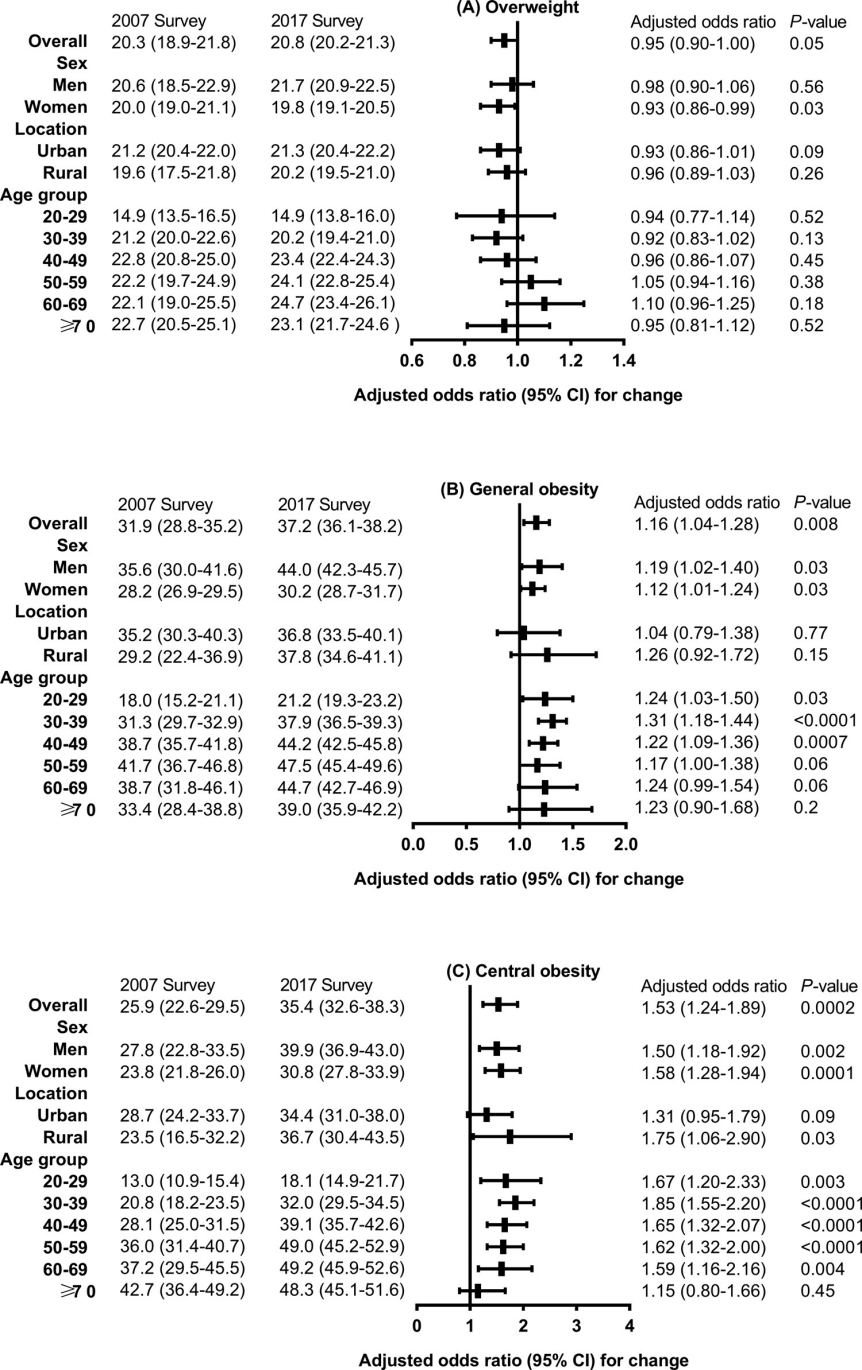
Changes in age- and sex-standardized prevalence of overweight, general obesity, and central obesity between 2007 and 2017 in adults in China. Values are weighted percentages (95% confidence intervals) unless stated otherwise. Logistic models were adjusted for age, sex, urbanization, ethnicity, income level, education level, and smoking status from 2007 to 2017.

**Figure 2:**
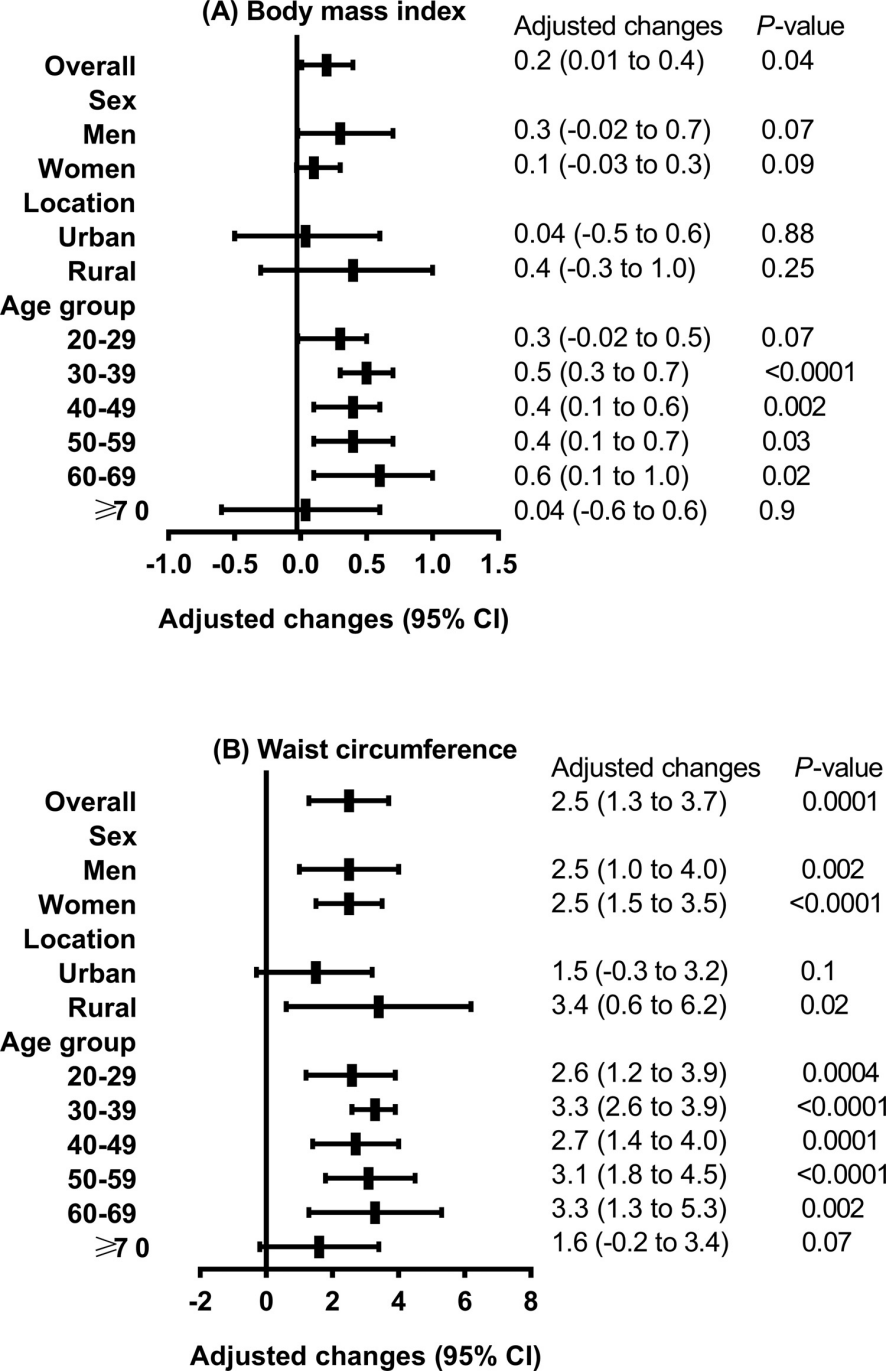
Adjusted increases in mean body mass index and waist circumference over the course of 10 years in adults in mainland China. Values are means (95% confidence intervals). Adjusted for age, sex, urbanization, ethnicity, income level, education level, and smoking status.

Figure 1 presents the changes in the age- and sex-standardized prevalence of overweight, general obesity, and central obesity in mainland China. Compared with 2007, the prevalence of general obesity (31.9% vs. 37.2%, *P*=0.008) and central obesity (25.9% vs. 35.4%, *P*=0.0002) was significantly higher in 2017 among the overall population. Figure 2 shows the changes in the mean BMI value and waist circumference between the first and second studies, with adjustment for age, sex, urbanization, ethnicity, income level, education level, and smoking status. Substantial increases in waist circumference were found consistently across all sex and age groups (except age≥70 years) and among rural residents (adjusted change, 3.4 cm; 95% CI, 0.6 to 6.2 cm; *P*=0.02). The mean BMI value (adjusted change, 0.2 kg/m^2^; 95% CI, 0.01 to 0.4 kg/m^2^; *P*=0.04) increased significantly in the overall population.

### 3.3 Changes in the Prevalence of Hypertension

**Figure 3:**
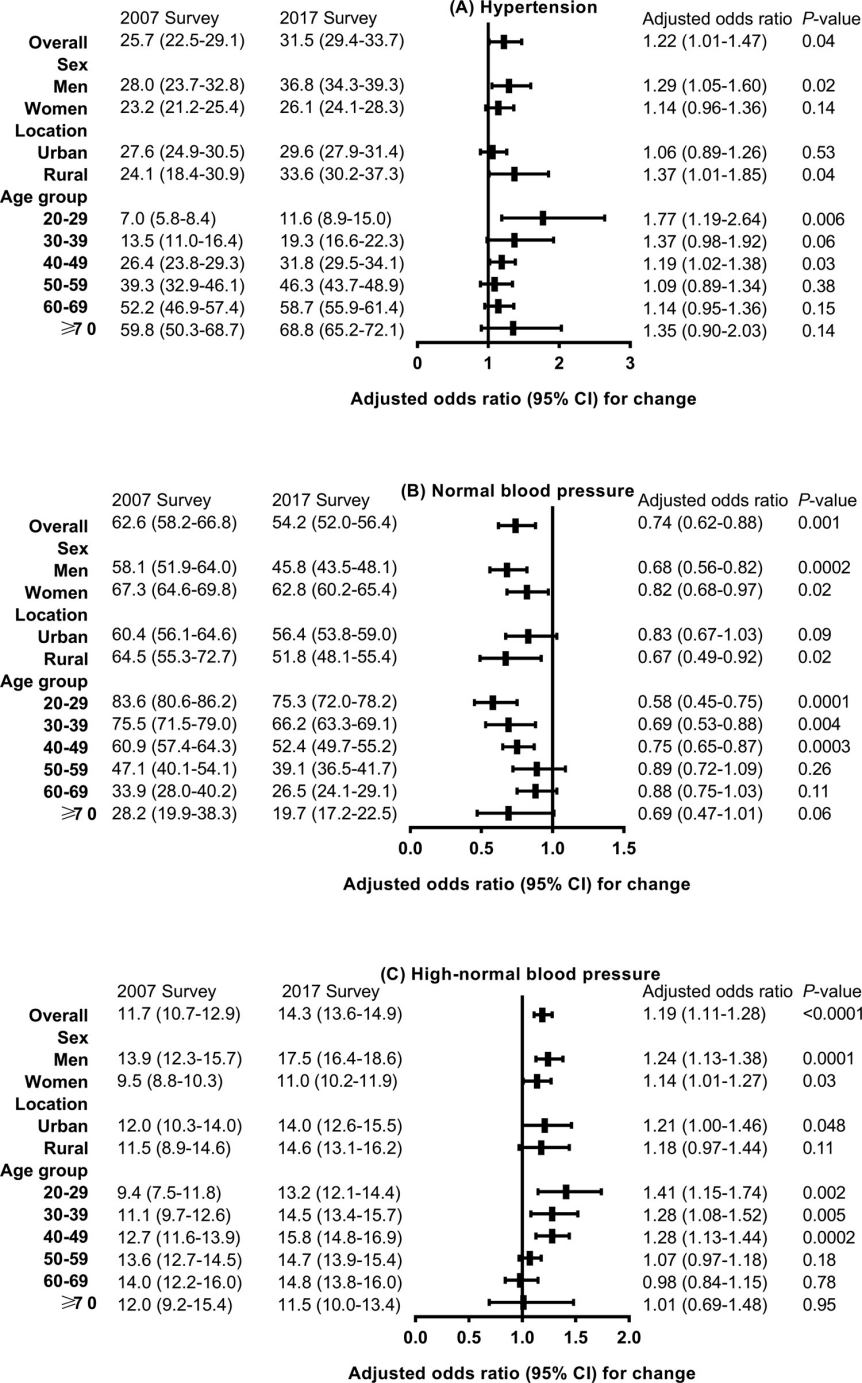
Changes in age- and sex-standardized prevalence of hypertension, normal blood pressure, and high-normal blood pressure between 2007 and 2017 in adults in China. Values are weighted percentages (95% confidence intervals) unless stated otherwise. Logistic models were adjusted for age, sex, urbanization, ethnicity, income level, education level, smoking status, body mass index, and waist circumference from 2007 to 2017.

Figure 3 presents the changes in the age- and sex-standardized prevalence of hypertension, normal blood pressure, and high-normal blood pressure in mainland China. Compared with 2007, the prevalence of hypertension (25.7% vs. 31.5%, *P*=0.04) and high-normal blood pressure (11.7% vs. 14.3%, *P*<0.0001) was higher, while the prevalence of normal blood pressure (62.6% vs. 54.2%, *P*=0.001) was lower among the overall population in 2017. A significantly higher prevalence of hypertension was seen in those aged 20-29 years (OR, 1.77; 95% CI: 1.19-2.64; *P*=0.006), men (OR, 1.29; 95% CI: 1.05-1.60; *P*=0.02), and rural residents (OR, 1.37; 95% CI: 1.01-1.85; *P*=0.04). No significantly increased prevalence of grade 1 or grade 2 hypertension was observed in the overall population (Supplementary Tables 1-2). For grade 1 hypertension, a significantly higher prevalence was seen only among participants aged 20 to 29 years (6.0% vs. 8.7%, *P*=0.002) in 2017. For grade 2 hypertension, a significantly higher prevalence was observed among participants aged 70 years and older (16.2% vs. 23.8%, *P*=0.046), among those who were overweight (6.1% vs. 8.5%, *P*=0.02), and among those without central obesity (5.1% vs. 7.3%, *P*=0.04) in 2017.

**Figure 4:**
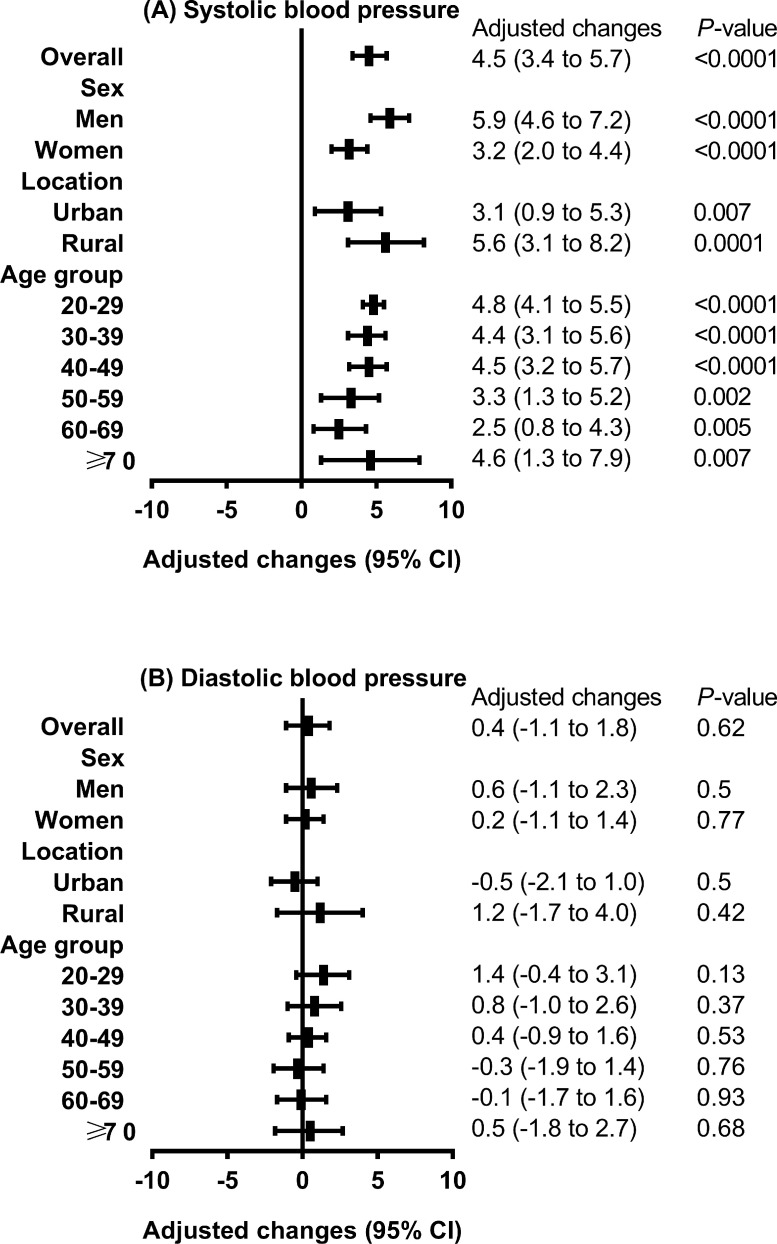
Adjusted increases in mean blood pressure over the course of 10 years in adults in mainland China. Values are means (95% confidence intervals). Adjusted for age, sex, urbanization, ethnicity, income level, education level, smoking status, body mass index, and waist circumference.

Figure 4 shows the change in the mean SBP and DBP stratified by sex, age group, and location between the two surveys, with adjustment for age, sex, urbanization, ethnicity, income level, education level, smoking status, BMI, and waist circumference. Significant increases were found consistently across all sexes, age groups, and regions for SBP (adjusted change, 4.5 mmHg; 95% CI, 3.4 to 5.7 mmHg, in the overall population), with greater increases among men (adjusted change, 5.9 mmHg; 95% CI, 4.6 to 7.2 mmHg; *P*<0.0001), participants aged 20-29 years (adjusted change, 4.8 mmHg; 95% CI, 4.1 to 5.5 mmHg; *P*<0.0001), and rural residents (adjusted change, 5.6 mmHg; 95% CI, 3.1 to 8.2 mmHg; *P*=0.0001).

### 3.4 Sensitivity Analysis

The results of the sensitivity analysis of the changes in the prevalence and mean BMI, waist circumference, and blood pressure levels are provided in the Supplement. The changes in the prevalence of overweight, general obesity, central obesity, hypertension, normal blood pressure, high-normal blood pressure, and grade 2 hypertension remained stable in three logistic regression models with adjustment for different numbers of demographic risk factors in the overall population (Supplementary Tables 1-8). In addition, the increases in mean BMI, waist circumference, and blood pressure levels remained stable in the two linear regression models after adjustment for different numbers of demographic risk factors in the overall population (Supplementary Tables 9-10).

## 4 Discussion

In the current large-sample, population-based cross-sectional study, we found that the age- and sex-standardized weighted prevalence of hypertension and obesity was higher among adults in 2017 in mainland China after adjusting for demographic confounding factors compared to that in 2007. In addition, significant increases in the mean BMI, waist circumference, and SBP occurred in adults over the decade after 2007. Moreover, a higher prevalence of high-normal blood pressure was observed in 2017 among men, urban residents, and young individuals. We used a nationally representative sample for large-scale recruitment, which could be generalized to adults aged 20 years and older in China.

Our study expands the existing literature on changes in obesity and hypertension in several ways. First, to our knowledge, our study is one of the largest to describe the changes in prevalence and blood pressure levels among adults in mainland China, which allowed us to explore associations across a variety of diverse subgroups. We found increasing changes in the prevalence of obesity and hypertension, which is consistent with previously reported changes in the Chinese population.[13,14] This is similar in India and Vietnam, whose prevalence of obesity and hypertension has shown upward trends.[24], [25], [26] However, the time-related trends in developing countries are different from those in Asian developed countries. The prevalence of hypertension in Japanese men dropped from 54.2% in 1980 to 50.1% in 2010, while that of women dropped from 47.4% to 37.8%.[27] In South Korea, the prevalence of hypertension in men dropped from 33.3% in 1998 to 30.3% in 2014 and in women dropped from 28.7% to 22.7%.[28] Furthermore, the United States, which also has a substantial burden of NCDs, has seen plateaus or even decreasing trends in the prevalence of obesity and hypertension in recent years.[29,30] In the past decade, rapid economic growth, which brought with it certain unhealthy lifestyles, especially a higher level of dietary sodium intake, is another new and crucial factor related to the increased prevalence of hypertension in China.[31] In addition, we found that young adults had a greater increase in SBP levels than somewhat older individuals. Trends in blood pressure levels in young adults are a marker of the future population burden of cardiovascular disease and may be particularly relevant in areas with high disease rates.[32] This phenomenon might be partially explained by the fact that in the Chinese population, the later the year in which an individual was born is, the higher their risk of developing hypertension is; while the development of the economy has gradually improved the standard of living of the Chinese people, the burdens of work and stress have increased.[13] Younger adults are somewhat more difficult to reach through traditional clinic-based preventive programs because they may be less aware of the long-term benefits of the early control of cardiovascular risk factors and are therefore less likely to be in contact with the health system and less motivated to make lifestyle changes.[33], [34], [35] Furthermore, the consumption of processed and packaged foods and beverages is on the rise among the young generation of China, and these types of foods usually contain higher levels of saturated fat, salt, and sugar.[36] Evidence from longitudinal studies has shown adverse effects of reductions in physical activity on weight change due to the use of occupational and household technology in China.[37], [38], [39] Young adults have increasingly entered the middle class in China, which might further expand the demand for convenient products that could reinforce unhealthy lifestyles.[40,41] In addition, given that the clinical importance of the treatment of hypertension in younger adults has been questioned in the past and that most previous studies of hypertension have focused on older individuals, there are limited recommendations for the management of hypertension in younger adults.[42], [43], [44] Thus, our findings may be a reflection of the lack of clinical data on this population and highlight the need for clinical trials in this population.

Second, our study is the first, to our knowledge, to describe the changes in the prevalence of high-normal blood pressure based on data from a national survey of the Chinese population. High-normal blood pressure is associated with increased risks of hypertension and cardiovascular disease and can be reduced through lifestyle modifications and the use of antihypertensive medication.[15] We found that the prevalence of high-normal blood pressure was higher in 2017 in the overall population. A previous study indicated that adults with prehypertension had risk factors for incident hypertension and had not made lifestyle modifications.[45] Importantly, low-cost interventions for preventing hypertension have been shown to be effective in all age groups, ethnicities, and sexes.[46] This indicates that there is a substantial opportunity to reduce the incidences of hypertension and cardiovascular disease through lifestyle changes. However, novel approaches for maintaining lifestyle modifications may be needed because increasing changes in central obesity and general obesity were also found in the current study. In addition, we found that the somewhat higher prevalence of hypertension and obesity shifted from urban to rural populations over the decade from 2007 to 2017. This result seems to be consistent with previous studies that found that the prevalence of central obesity of residents in rural areas increased more rapidly than that of residents in urban areas.[47,48] These findings may be related to the changes in socioeconomic structure led by urbanization in China.[48] A previous observation confirmed that chronic health conditions are related to modernization and affluence and that the emergence of these problems is no longer limited to urban populations.[49] The per capita food consumption of Chinese rural households increased by 2.6 times from 1997 to 2012. The Engel coefficient of urban and rural households dropped by 10.4 and 15.8 percentage points from 1997 to 2012, respectively.[50] With the advancement of urbanization, the food consumption ability of rural adults has developed rapidly. Therefore, high-fat diets and reduced physical activity may exacerbate health deterioration, such as the higher prevalence of obesity in more urbanized regions.[51] In addition, high sodium intake was associated with a higher risk of central obesity than general obesity.[52] Significant differences in the prevalence of central obesity among rural residents may be due to increasingly high dietary sodium in rural areas in China.[53] China has a large rural population, and sanitation is lacking in rural areas; thus, an increased prevalence of obesity and hypertension in rural areas will lead to increased incidences of NCDs. Given the greater increases in SBP and waist circumference in rural populations, a large number of people are at risk of developing hypertension in the absence of the implementation of effective preventive measures.

Several recommendations for national policies and efforts may potentially combat the further development of obesity and hypertension in China.[12] First, to establish fiscal policies to prevent and control obesity and levy taxes on unhealthy foods and beverages, subsidies should be provided to promote healthy diets and healthy lifestyles. Second, activity centres, indoor and outdoor fitness venues, and self-service health management inspection points equipped with height, weight, and blood pressure measurements should be established. Third, obesity treatment should be included in the coverage of health insurance, and medical expense reimbursement should be correlated with the results of weight management of obese patients. Fourth, obesity prevention policies and strategies should take the inequalities found in this study into full consideration and be tailored to high-risk groups to prevent a further gap in obesity prevalence among subgroups and ensure health equity.

The 2007 and 2017 studies have potential limitations, some of which have been mentioned in previous studies.[17,18] First, they did not assess dietary intake, alcohol consumption, and physical activity. Therefore, we were not able to determine the associations between these factors and the changes in the prevalence of obesity and hypertension. Second, the 2020 ISH guidelines recommend longitudinal and three measurements of blood pressure levels for the diagnosis of hypertension.[15] Because the two studies were large-scale population-based cross-sectional surveys, blood pressure was only measured in the participants two times in a single day. Considering the effect of regression to the mean, this may have overestimated the prevalence of hypertension. However, the effect of regression on the mean should not be substantial. Third, although the survey staff were highly trained, their efficacy or skill level may have resulted in some misclassification errors. The limitations of the current analysis also warrant discussion. The first and second surveys used different types of blood pressure monitoring, which would produce systematic error, although previous studies have proven good agreement for blood pressure measurements between mercury sphygmomanometers and electronic devices.[54,55]

In conclusion, the prevalence of hypertension, high-normal blood pressure, and obesity was significantly higher among adults in mainland China after adjustment for demographic confounding factors in 2017. The BMI, waist circumference, and SBP levels increased slightly, with a greater increase in SBP in men, young adults, and rural residents. More targeted interventions and prevention strategies are needed to offset the increasing risk of cardiovascular disease due to increases in the prevalence of hypertension and obesity.

## Author Contributions

Yaxin Lai, Zhongyan Shan, Weiping Teng and Yongze Li had full access to all the data in the study and take responsibility for the integrity of the data and the accuracy of the data analysis.

Concept and design: Yaxin Lai, Zhongyan Shan, Weiping Teng.

Acquisition, analysis, and interpretation of the data: Yaxin Lai, Zhongyan Shan, Weiping Teng and Yongze Li.

Drafting of the manuscript: Yongze Li.

Statistical analysis: Yongze Li.

Obtaining funding: Zhongyan Shan and Weiping Teng.

Administrative, technical and material support: All authors.

Study supervision: Zhongyan Shan, Weiping Teng, Yongze Li, Yaxin Lai, Di Teng, Xiaochun Teng, Xiaoguang Shi.

## Declaration of Competing Interest

The authors declare no conflict of interests.

## Appendix Supplementary materials

Image, application 1
