# Supplementary material for: Changes in the prevalence of obesity and hypertension and demographic risk factor profiles in China over 10 years: two national cross-sectional surveys
Source: Lancet Reg Health West Pac. 2021 Jul 31;15:100227. doi: 10.1016/j.lanwpc.2021.100227 (PMC8342963; doi:10.1016/j.lanwpc.2021.100227)
Supplement: Supplementary file 1 [file mmc1.docx]

**SUPPLEMENTAL MATERIAL**

**Supplementary Figure 1. Flowchart depicting survey design**

Supplementary Figure Legends: For sampling process of the China National Diabetes and Metabolic Disorder Study, in the first-stage, 12 provinces and autonomous regions were selected from all 6 geographic regions in mainland China, in addition to the municipalities of Beijing and Shanghai. One midsize city (population 200,000-1,000,000), one developed and one underdeveloped county, which were at approximately the 67th and 33rd percentiles of gross domestic product (GDP) per capita among all counties within each province, respectively, were selected, plus the provincial capitals. In the second-stage, one to four urban districts from Beijing, Shanghai, and each provincial capital and a midsize city were randomly selected (a total of 76 urban districts). Two rural districts were randomly selected from each village and 4 rural districts were randomly selected from the Beijing and Shanghai countryside (a total of 56 rural districts). In the third-stage, two urban residential communities or rural residential communities were randomly selected from each urban city district and rural township district, respectively. At the final stage, eligible individuals from the local resident registration list who met the inclusion criteria were randomly selected according to age-sex-location composition among populations from China’s 2006 national census data. For sampling process of the Thyroid Disorders, Iodine Status and Diabetes Epidemiological Survey, at the first stage, one city was selected from each province in all 31 provinces of mainland China. Finally, 31 cities were selected and divided into developed, developing and underdeveloped cities, based on gross domestic product per capita, concentration of commercial resources, the extent to which a city serves as a commercial hub, vitality of residents, diversity of lifestyle and future dynamism. One county was randomly selected from each city. At the second stage, one urban district was randomly selected from each city (a total of 31 urban districts) and one rural district was randomly selected from each village (a total of 31 rural districts). At the third stage, two urban residential communities or rural residential communities were randomly selected from each urban city district and rural township district, respectively. At the final stage, eligible individuals from the local resident registration list who met the inclusion criteria were randomly selected according to age-sex-location composition among the population from China’s 2010 national census data.


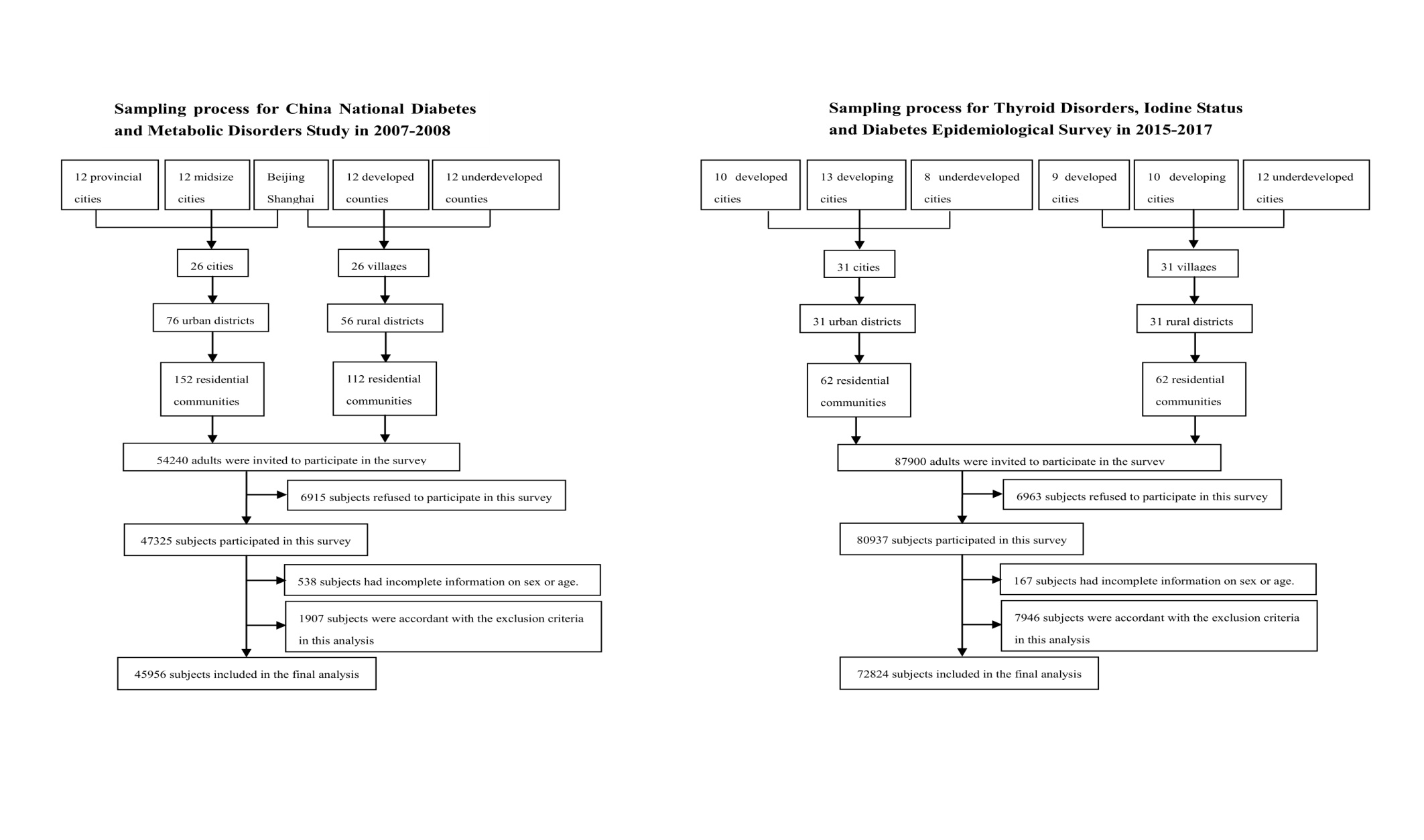


**Supplementary Table 1. Changes in age- and sex-standardized prevalence of overweight between 2007 and 2017 in adults in China by subgroups.**

|  | Prevalence (95% CI) | | Odds ratio (95% CI) for change from 2007 to 2017 | | |
| --- | --- | --- | --- | --- | --- |
|  | 2007 | 2017 | Model 1^a^ | Model 2^b^ | Model 3^c^ |
| Overall | 20.3 (18.9-21.8) | 20.8 (20.2-21.3) | 1.01 (0.91-1.11) | 1.02 (0.92-1.13) | 0.95 (0.90-1.00) |
| Sex | | | | | |
| Men | 20.6 (18.5-22.9) | 21.7 (20.9-22.5) | 1.05 (0.91-1.20) | 1.06 (0.92-1.22) | 0.98 (0.90-1.06) |
| Women | 20.0 (19.0-21.1) | 19.8 (19.1-20.5) | 0.97 (0.89-1.06) | 0.98 (0.90-1.07) | 0.93 (0.86-0.99)^*^ |
| Location | | | | | |
| Urban | 21.2 (20.4-22.0) | 21.3 (20.4-22.2) | 0.97 (0.90-1.04) | 0.99 (0.92-1.07) | 0.93 (0.86-1.01) |
| Rural | 19.6 (17.5-21.8) | 20.2 (19.5-21.0) | 1.04 (0.91-1.20) | 1.04 (0.91-1.20) | 0.96 (0.89-1.03) |
| Age group | | | | | |
| 20-29 | 14.9 (13.5-16.5) | 14.9 (13.8-16.0) | 1.00 (0.86-1.15) | 1.04 (0.91-1.21) | 0.94 (0.77-1.14) |
| 30-39 | 21.2 (20.0-22.6) | 20.2 (19.4-21.0) | 0.94 (0.86-1.03) | 0.94 (0.86-1.04) | 0.92 (0.83-1.02) |
| 40-49 | 22.8 (20.8-25.0) | 23.4 (22.4-24.3) | 1.03 (0.90-1.17) | 1.03 (0.90-1.18) | 0.96 (0.86-1.07) |
| 50-59 | 22.2 (19.7-24.9) | 24.1 (22.8-25.4) | 1.11 (0.94-1.31) | 1.11 (0.94-1.31) | 1.05 (0.94-1.16) |
| 60-69 | 22.1 (19.0-25.5) | 24.7 (23.4-26.1) | 1.16 (0.95-1.42) | 1.16 (0.95-1.42) | 1.10 (0.96-1.25) |
| ≥70 | 22.7 (20.5-25.1) | 23.1 (21.7-24.6 ) | 1.02 (0.88-1.19) | 1.03 (0.86-1.23) | 0.95 (0.81-1.12) |
| Ethnicity | | | | | |
| Han | 21.2 (20.5-21.8) | 20.8 (20.3-21.4) | 0.96 (0.92-1.01) | 0.97 (0.92-1.02) | 0.93 (0.89-0.98)^*^ |
| Non-Han | 14.8 (12.3-17.8) | 18.8 (16.2-21.6) | 1.32 (1.00-1.74) | 1.32 (1.00-1.76) | 1.12 (0.94-1.32) |
| Income per year | | | | | |
| ≤30000 Yuan | 19.8 (17.9-21.7) | 19.8 (18.8-20.8) | 1.00 (0.87-1.14) | 0.98 (0.86-1.12) | 0.96 (0.89-1.04) |
| >30000 Yuan | 22.9 (21.8-24.0) | 21.7 (21.0-22.5) | 0.92 (0.86-0.99)^*^ | 0.94 (0.87-1.02) | 0.94 (0.87-1.01) |
| Education | | | | | |
| Less than college | 20.0 (18.1-22.0) | 20.6 (19.9-21.3) | 1.06 (0.93-1.20) | 1.04 (0.92-1.18) | 0.98 (0.92-1.04) |
| College and above | 22.0 (21.0-23.1) | 21.7 (20.9-22.6) | 0.93 (0.84-1.02) | 0.98 (0.89-1.09) | 0.91 (0.83-0.99)^*^ |
| Cigarette smoking | | | | | |
| Current non-smoker | 20.7 (19.7-21.9) | 21.2 (20.6-21.9) | 1.00 (0.93-1.08) | 1.01 (0.94-1.09) | 0.95 (0.90-1.00) |
| Current smoker | 19.4 (16.7-22.4) | 17.9 (16.6-19.2) | 1.04 (0.85-1.27) | 1.05 (0.85-1.29) | 0.95 (0.85-1.06) |

^a^ Model 1: Unadjusted.

^b^ Model 2: Adjusted for age and sex.

^c^ Model 3: Adjusted for age, sex, location, ethnicity, income level, education level, and smoking status.

^*^ indicates a *P*-value less than 0.05.

**Supplementary Table 2. Changes in age- and sex-standardized prevalence of general obesity between 2007 and 2017 in adults in China by subgroups.**

|  | Prevalence (95% CI) | | Odds ratio (95% CI) for change from 2007 to 2017 | | |
| --- | --- | --- | --- | --- | --- |
|  | 2007 | 2017 | Model 1^a^ | Model 2^b^ | Model 3^c^ |
| Overall | 31.9 (28.8-35.2) | 37.2 (36.1-38.2) | 1.22 (1.05-1.42)^*^ | 1.25 (1.07-1.46)^*^ | 1.16 (1.04-1.28)^*^ |
| Sex | | | | | |
| Men | 35.6 (30.0-41.6) | 44.0 (42.3-45.7) | 1.39 (1.07-1.80)^*^ | 1.40 (1.08-1.82)^*^ | 1.19 (1.02-1.40)^*^ |
| Women | 28.2 (26.9-29.5) | 30.2 (28.7-31.7) | 1.06 (0.95-1.17) | 1.08 (0.98-1.20) | 1.12 (1.01-1.24)^*^ |
| Location | | | | | |
| Urban | 35.2 (30.3-40.3) | 36.8 (33.5-40.1) | 1.02 (0.77-1.35) | 1.07 (0.81-1.42) | 1.04 (0.79-1.38) |
| Rural | 29.2 (22.4-36.9) | 37.8 (34.6-41.1) | 1.44 (0.98-2.12) | 1.44 (0.98-2.13) | 1.26 (0.92-1.72) |
| Age group | | | | | |
| 20-29 | 18.0 (15.2-21.1) | 21.2 (19.3-23.2) | 1.24 (0.99-1.56) | 1.34 (1.06-1.68)^*^ | 1.24 (1.03-1.50)* |
| 30-39 | 31.3 (29.7-32.9) | 37.9 (36.5-39.3) | 1.36 (1.24-1.49)^*^ | 1.38 (1.25-1.52)^*^ | 1.31 (1.18-1.44)* |
| 40-49 | 38.7 (35.7-41.8) | 44.2 (42.5-45.8) | 1.25 (1.09-1.44)^*^ | 1.26 (1.09-1.45)^*^ | 1.22 (1.09-1.36)* |
| 50-59 | 41.7 (36.7-46.8) | 47.5 (45.4-49.6) | 1.26 (1.01-1.58)^*^ | 1.27 (1.02-1.59)^*^ | 1.17 (1.00-1.38) |
| 60-69 | 38.7 (31.8-46.1) | 44.7 (42.7-46.9) | 1.29 (0.94-1.76) | 1.29 (0.94-1.77) | 1.24 (0.99-1.54) |
| ≥70 | 33.4 (28.4-38.8) | 39.0 (35.9-42.2) | 1.28 (0.98-1.67) | 1.31 (1.01-1.70)^*^ | 1.23 (0.90-1.68) |
| Ethnicity | | | | | |
| Han | 33.9 (31.9-35.9) | 37.4 (36.3-38.5) | 1.13 (1.01-1.26)^*^ | 1.15 (1.04-1.28)^*^ | 1.12 (1.00-1.25)* |
| Non-Han | 19.2 (11.6-30.0) | 31.5 (25.8-37.7) | 1.89 (1.02-3.50)^*^ | 1.90 (1.01-3.56)^*^ | 1.49 (0.97-2.29) |
| Income per year | | | | | |
| ≤30000 Yuan | 31.2 (27.4-35.3) | 37.1 (35.4-38.8) | 1.28 (1.04-1.57)^*^ | 1.25 (1.02-1.53)^*^ | 1.21 (1.05-1.40)* |
| >30000 Yuan | 35.7 (34.1-37.4) | 37.1 (35.6-38.7) | 1.01 (0.91-1.13) | 1.07 (0.96-1.20) | 1.06 (0.95-1.18) |
| Education | | | | | |
| Less than college | 31.9 (27.8-36.3) | 39.3 (38.0-40.6) | 1.39 (1.13-1.70)^*^ | 1.36 (1.11-1.67)^*^ | 1.25 (1.10-1.43)* |
| College and above | 32.5 (29.6-35.5) | 34.3 (32.3-36.4) | 0.99 (0.86-1.13) | 1.13 (0.98-1.31) | 1.05 (0.91-1.22) |
| Cigarette smoking | | | | | |
| Current non-smoker | 33.9 (31.7-36.2) | 37.1 (36.0-38.2) | 1.08 (0.98-1.20) | 1.12 (1.02-1.23)^*^ | 1.08 (0.99-1.18) |
| Current smoker | 28.4 (21.0-37.2) | 40.6 (38.1-43.1) | 1.68 (1.18-2.38)^*^ | 1.67 (1.18-2.38)^*^ | 1.40 (1.15-1.71)^*^ |

^a^ Model 1: Unadjusted.

^b^ Model 2: Adjusted for age and sex.

^c^ Model 3: Adjusted for age, sex, location, ethnicity, income level, education level, and smoking status.

^*^ indicates a *P*-value less than 0.05.

**Supplementary Table 3. Changes in age- and sex-standardized prevalence of central obesity between 2007 and 2017 in adults in China by subgroups.**

|  | Prevalence (95% CI) | | Odds ratio (95% CI) for change from 2007 to 2017 | | |
| --- | --- | --- | --- | --- | --- |
|  | 2007 | 2017 | Model 1^a^ | Model 2^b^ | Model 3^c^ |
| Overall | 25.9 (22.6-29.5) | 35.4 (32.6-38.3) | 1.52 (1.22 to 1.90)^*^ | 1.60 (1.28 to 2.01)^*^ | 1.53 (1.24-1.89)^*^ |
| Sex | | | | | |
| Men | 27.8 (22.8-33.5) | 39.9 (36.9-43.0) | 1.68 (1.24 to 2.27)^*^ | 1.73 (1.27 to 2.34)^*^ | 1.50 (1.18-1.92)^*^ |
| Women | 23.8 (21.8-26.0) | 30.8 (27.8-33.9) | 1.37 (1.12 to 1.66)^*^ | 1.47 (1.20 to 1.79)^*^ | 1.58 (1.28-1.94)^*^ |
| Location | | | | | |
| Urban | 28.7 (24.2-33.7) | 34.4 (31.0-38.0) | 1.22 (0.91 to 1.65) | 1.34 (0.99 to 1.82) | 1.31 (0.95-1.79) |
| Rural | 23.5 (16.5-32.2) | 36.7 (30.4-43.5) | 1.88 (1.12 to 3.15)^*^ | 1.90 (1.11 to 3.25)^*^ | 1.75 (1.06-2.90)^*^ |
| Age group | | | | | |
| 20-29 | 13.0 (10.9-15.4) | 18.1 (14.9-21.7) | 1.49 (1.10 to 2.02)^*^ | 1.60 (1.19 to 2.16)^*^ | 1.67 (1.20-2.33)^*^ |
| 30-39 | 20.8 (18.2-23.5) | 32.0 (29.5-34.5) | 1.82 (1.50 to 2.21)^*^ | 1.87 (1.53 to 2.28)^*^ | 1.85 (1.55-2.20)^*^ |
| 40-49 | 28.1 (25.0-31.5) | 39.1 (35.7-42.6) | 1.64 (1.32 to 2.03)^*^ | 1.66 (1.33 to 2.06)^*^ | 1.65 (1.32-2.07)^*^ |
| 50-59 | 36.0 (31.4-40.7) | 49.0 (45.2-52.9) | 1.71 (1.33 to 2.21)^*^ | 1.71 (1.32 to 2.20)^*^ | 1.62 (1.32-2.00)^*^ |
| 60-69 | 37.2 (29.5-45.5) | 49.2 (45.9-52.6) | 1.64 (1.13 to 2.38)^*^ | 1.65 (1.13 to 2.41)^*^ | 1.59 (1.16-2.16)^*^ |
| ≥70 | 42.7 (36.4-49.2) | 48.3 (45.1-51.6) | 1.26 (0.94 to 1.69) | 1.28 (0.97 to 1.69) | 1.15 (0.80-1.66) |
| Ethnicity | | | | | |
| Han | 27.4 (25.5-29.4) | 35.5 (32.6-38.5) | 1.40 (1.18 to 1.67)^*^ | 1.48 (1.25 to 1.76)^*^ | 1.47 (1.20-1.79)^*^ |
| Non-Han | 15.6 (8.8-26.2) | 33.1 (28.5-38.1) | 2.66 (1.38 to 5.13)^*^ | 2.75 (1.36 to 5.56)^*^ | 2.46 (1.36-4.42)^*^ |
| Income per year | | | | | |
| ≤30000 Yuan | 25.2 (21.4-29.5) | 36.2 (31.6-41.2) | 1.72 (1.28 to 2.32)^*^ | 1.66 (1.22 to 2.26)^*^ | 1.61 (1.24-2.10)^*^ |
| >30000 Yuan | 28.3 (26.6-30.0) | 34.7 (32.8-36.5) | 1.27 (1.11 to 1.46)^*^ | 1.39 (1.21 to 1.59)^*^ | 1.36 (1.20-1.55)^*^ |
| Education | | | | | |
| Less than college | 25.7 (21.8-30.0) | 37.3 (34.6-40.1) | 1.77 (1.38 to 2.28)^*^ | 1.73 (1.34 to 2.24)^*^ | 1.66 (1.33-2.07)^*^ |
| College and above | 26.5 (24.4-28.6) | 32.1 (29.3-34.9) | 1.18 (0.96 to 1.44) | 1.39 (1.13 to 1.71)^*^ | 1.36 (1.06-1.73)^*^ |
| Cigarette smoking | | | | | |
| Current non-smoker | 26.8 (24.1-29.6) | 34.7 (31.7-37.8) | 1.39 (1.13 to 1.70)^*^ | 1.49 (1.22 to 1.81)^*^ | 1.48 (1.20-1.83)^*^ |
| Current smoker | 26.2 (21.0-32.1) | 38.6 (35.2-42.1) | 1.95 (1.35 to 2.82)^*^ | 1.97 (1.35 to 2.87)^*^ | 1.71 (1.31-2.22)^*^ |

^a^ Model 1: Unadjusted.

^b^ Model 2: Adjusted for age and sex.

^c^ Model 3: Adjusted for age, sex, location, ethnicity, income level, education level, and smoking status.

^*^ indicates a *P*-value less than 0.05.

**Supplementary Table 4. Changes in age- and sex-standardized prevalence of hypertension between 2007 and 2017 in adults in China by subgroups.**

|  | Prevalence (95% CI) | | Odds ratio (95% CI) for change from 2007 to 2017 | | |
| --- | --- | --- | --- | --- | --- |
|  | 2007 | 2017 | Model 1^a^ | Model 2^b^ | Model 3^c^ |
| Overall | 25.7 (22.5-29.1) | 31.5 (29.4-33.7) | 1.27 (1.02 to 1.56)* | 1.39 (1.10 to 1.76)* | 1.22 (1.01-1.47)* |
| Sex | | | | | |
| Men | 28.0 (23.7-32.8) | 36.8 (34.3-39.3) | 1.41 (1.09 to 1.82)* | 1.55 (1.17 to 2.05)* | 1.29 (1.05-1.60)* |
| Women | 23.2 (21.2-25.4) | 26.1 (24.1-28.3) | 1.12 (0.92 to 1.36) | 1.20 (0.97 to 1.48) | 1.14 (0.96-1.36) |
| Location | | | | | |
| Urban | 27.6 (24.9-30.5) | 29.6 (27.9-31.4) | 0.98 (0.82 to 1.17) | 1.12 (0.91 to 1.37) | 1.06 (0.89-1.26) |
| Rural | 24.1 (18.4-30.9) | 33.6 (30.2-37.3) | 1.62 (1.11 to 2.38)* | 1.71 (1.10 to 2.64)* | 1.37 (1.01-1.85)* |
| Age group | | | | | |
| 20-29 | 7.0 (5.8-8.4) | 11.6 (8.9-15.0) | 1.75 (1.23 to 2.50)* | 1.84 (1.29 to 2.64)* | 1.77 (1.19-2.64)* |
| 30-39 | 13.5 (11.0-16.4) | 19.3 (16.6-22.3) | 1.56 (1.16 to 2.10)* | 1.60 (1.18 to 2.19)* | 1.37 (0.98-1.92) |
| 40-49 | 26.4 (23.8-29.3) | 31.8 (29.5-34.1) | 1.30 (1.09 to 1.55)* | 1.31 (1.10 to 1.57)* | 1.19 (1.02-1.38)* |
| 50-59 | 39.3 (32.9-46.1) | 46.3 (43.7-48.9) | 1.33 (0.99 to 1.79) | 1.30 (0.97 to 1.75) | 1.09 (0.89-1.34) |
| 60-69 | 52.2 (46.9-57.4) | 58.7 (55.9-61.4) | 1.30 (1.02 to 1.65)* | 1.31 (1.03 to 1.66)* | 1.14 (0.95-1.36) |
| ≥70 | 59.8 (50.3-68.7) | 68.8 (65.2-72.1) | 1.48 (0.97 to 2.25) | 1.48 (0.97 to 2.26) | 1.35 (0.90-2.03) |
| Ethnicity | | | | | |
| Han | 27.3 (25.2-29.6) | 31.9 (29.7-34.1) | 1.17 (0.99 to 1.38) | 1.28 (1.07 to 1.53)* | 1.21 (1.00-1.47)* |
| Non-Han | 14.6 (10.3-20.4) | 23.3 (19.8-27.2) | 1.77 (1.22 to 2.57)* | 1.90 (1.16 to 3.12)* | 1.21 (0.90-1.64) |
| Income per year | | | | | |
| ≤30000 Yuan | 25.3 (22.0-29.0) | 32.2 (29.6-34.9) | 1.52 (1.19 to 1.95)* | 1.44 (1.11 to 1.86)* | 1.21 (1.01-1.45)* |
| >30000 Yuan | 26.6 (23.1-30.3) | 31.1 (29.1-33.1) | 1.13 (0.89 to 1.43) | 1.31 (1.01 to 1.69)* | 1.23 (0.94-1.60) |
| Education | | | | | |
| Less than college | 25.7 (22.1-29.7) | 33.3 (30.6-36.1) | 1.53 (1.20 to 1.94)* | 1.47 (1.13 to 1.92)* | 1.23 (1.00-1.51) |
| College and above | 25.8 (24.1-27.6) | 28.3 (26.5-30.2) | 1.00 (0.81 to 1.24) | 1.26 (1.07 to 1.49)* | 1.22 (0.98-1.53) |
| Cigarette smoking | | | | | |
| Current non-smoker | 27.1 (24.5-29.8) | 31.7 (29.7-33.7) | 1.14 (0.94 to 1.39) | 1.27 (1.04 to 1.54)* | 1.19 (1.00-1.42) |
| Current smoker | 21.8 (16.6-28.2) | 32.6 (29.4-35.9) | 1.65 (1.22 to 2.23)* | 1.76 (1.24 to 2.48)* | 1.31 (1.04-1.65)* |
| Body mass index | | | | | |
| <23 | 15.4 (12.6-18.8) | 21.5 (19.7-23.5) | 1.30 (1.01-1.67)* | 1.56 (1.16-2.11)* | 1.47 (1.16-1.86)* |
| 23-<25 | 25.1 (22.4-28.0) | 30.3 (28.0-32.8) | 1.28 (1.03-1.58)* | 1.33 (1.06-1.68)* | 1.26 (0.98-1.61) |
| ≥25 | 38.9 (36.8-41.1) | 41.5 (38.9-44.0) | 1.11 (0.95-1.30) | 1.15 (0.98-1.35) | 1.08 (0.91-1.28) |
| Waist circumference | | | | | |
| Men <90cm, women <80 cm | 20.1 (17.0-23.5) | 25.7 (23.7-27.8) | 1.26 (0.99 to 1.58) | 1.44 (1.11 to 1.86)* | 1.37 (1.11-1.69)* |
| Men ≥90 cm, women ≥80 cm | 39.8 (37.3-42.4) | 41.1 (38.0-44.2) | 0.98 (0.82 to 1.18) | 1.05 (0.87 to 1.26) | 1.04 (0.85-1.26) |

^a^ Model 1: Unadjusted.

^b^ Model 2: Adjusted for age and sex.

^c^ Model 3: Adjusted for age, sex, location, ethnicity, income level, education level, and smoking status, BMI, and waist circumference.

^*^ indicates a *P*-value less than 0.05.

**Supplementary Table 5. Changes in age- and sex-standardized prevalence of normal blood pressure between 2007 and 2017 in adults in China by subgroups.**

|  | Prevalence (95% CI) | | Odds ratio (95% CI) for change from 2007 to 2017 | | |
| --- | --- | --- | --- | --- | --- |
|  | 2007 | 2017 | Model 1^a^ | Model 2^b^ | Model 3^c^ |
| Overall | 62.6 (58.2-66.8) | 54.2 (52.0-56.4) | 0.74 (0.60 to 0.92)* | 0.66 (0.52 to 0.84)* | 0.74 (0.62-0.88)* |
| Sex | | | | | |
| Men | 58.1 (51.9-64.0) | 45.8 (43.5-48.1) | 0.64 (0.49 to 0.84)* | 0.58 (0.44 to 0.78)* | 0.68 (0.56-0.82)* |
| Women | 67.3 (64.6-69.8) | 62.8 (60.2-65.4) | 0.86 (0.71 to 1.05) | 0.78 (0.63 to 0.97)* | 0.82 (0.68-0.97)* |
| Location | | | | | |
| Urban | 60.4 (56.1-64.6) | 56.4 (53.8-59.0) | 0.94 (0.77 to 1.16) | 0.82 (0.64 to 1.05) | 0.83 (0.67-1.03) |
| Rural | 64.5 (55.3-72.7) | 51.8 (48.1-55.4) | 0.58 (0.39 to 0.88)* | 0.54 (0.34 to 0.87)* | 0.67 (0.49-0.92)* |
| Age group | | | | | |
| 20-29 | 83.6 (80.6-86.2) | 75.3 (72.0-78.2) | 0.59 (0.46 to 0.77)* | 0.56 (0.43 to 0.74)* | 0.58 (0.45-0.75)* |
| 30-39 | 75.5 (71.5-79.0) | 66.2 (63.3-69.1) | 0.63 (0.49 to 0.80)* | 0.61 (0.47 to 0.79)* | 0.69 (0.53-0.88)* |
| 40-49 | 60.9 (57.4-64.3) | 52.4 (49.7-55.2) | 0.71 (0.59 to 0.84)* | 0.70 (0.58 to 0.83)* | 0.75 (0.65-0.87)* |
| 50-59 | 47.1 (40.1-54.1) | 39.1 (36.5-41.7) | 0.72 (0.53 to 0.97)* | 0.73 (0.54 to 0.99)* | 0.89 (0.72-1.09) |
| 60-69 | 33.9 (28.0-40.2) | 26.5 (24.1-29.1) | 0.70 (0.52 to 0.95)* | 0.70 (0.52 to 0.95)* | 0.88 (0.75-1.03) |
| ≥70 | 28.2 (19.9-38.3) | 19.7 (17.2-22.5) | 0.62 (0.38 to 1.02) | 0.63 (0.40 to 1.01) | 0.69 (0.47-1.01) |
| Ethnicity | | | | | |
| Han | 60.3 (57.6-62.9) | 53.7 (51.5-56.0) | 0.81 (0.69 to 0.95)* | 0.73 (0.61 to 0.86)* | 0.75 (0.62-0.89)* |
| Non-Han | 77.9 (72.4-82.7) | 65.2 (58.5-71.4) | 0.54 (0.39 to 0.74)* | 0.50 (0.31 to 0.78)* | 0.72 (0.56-0.94)* |
| Income per year | | | | | |
| ≤30000 Yuan | 63.1 (58.2-67.9) | 53.6 (50.9-56.3) | 0.63 (0.49 to 0.82)* | 0.64 (0.49 to 0.84)* | 0.75 (0.63-0.90)* |
| >30000 Yuan | 62.1 (58.3-65.7) | 54.8 (52.7-56.8) | 0.80 (0.65 to 0.98)* | 0.68 (0.54 to 0.85)* | 0.72 (0.58-0.90)* |
| Education | | | | | |
| Less than college | 62.3 (57.0-67.4) | 52.4 (49.7-55.1) | 0.62 (0.48 to 0.80)* | 0.63 (0.48 to 0.84)* | 0.76 (0.62-0.94)* |
| College and above | 62.9 (60.7-65.0) | 57.7 (55.4-59.9) | 0.86 (0.71 to 1.03) | 0.68 (0.58 to 0.81)* | 0.68 (0.55-0.84)* |
| Cigarette smoking | | | | | |
| Current non-smoker | 60.8 (57.4-64.2) | 54.0 (51.8-56.2) | 0.83 (0.68 to 1.01) | 0.73 (0.60 to 0.89)* | 0.76 (0.64-0.91)* |
| Current smoker | 67.0 (59.6-73.6) | 53.0 (49.5-56.4) | 0.55 (0.40 to 0.75)* | 0.52 (0.36 to 0.73)* | 0.68 (0.55-0.84)* |
| Body mass index | | | | | |
| <23 | 75.7 (71.3-79.7) | 67.3 (64.7-69.9) | 0.76 (0.59-0.97)* | 0.63 (0.48-0.84)* | 0.66 (0.53-0.81)* |
| 23-<25 | 64.1 (60.5-67.6) | 57.0 (54.7-59.3) | 0.76 (0.62-0.93)* | 0.73 (0.59-0.90)* | 0.78 (0.64-0.94)* |
| ≥25 | 48.9 (46.3-51.6) | 44.8 (42.2-47.5) | 0.84 (0.70-1.01) | 0.82 (0.68-0.98)* | 0.86 (0.71-1.03) |
| Waist circumference | | | | | |
| Men <90cm, women <80 cm | 68.9 (64.2-73.3) | 60.6 (58.3-62.9) | 0.76 (0.61 to 0.95)* | 0.65 (0.51 to 0.84)* | 0.67 (0.56-0.81)* |
| Men ≥90 cm, women ≥80 cm | 45.3 (42.6-48.1) | 42.3 (39.0-45.7) | 0.95 (0.78 to 1.15) | 0.89 (0.74 to 1.07) | 0.89 (0.74-1.07) |

^a^ Model 1: Unadjusted.

^b^ Model 2: Adjusted for age and sex.

^c^ Model 3: Adjusted for age, sex, location, ethnicity, income level, education level, and smoking status, BMI, and waist circumference.

^*^ indicates a *P*-value less than 0.05.

**Supplementary Table 6. Changes in age- and sex-standardized prevalence of high-normal blood pressure between 2007 and 2017 in adults in China by subgroups.**

|  | Prevalence (95% CI) | | Odds ratio (95% CI) for change from 2007 to 2017 | | |
| --- | --- | --- | --- | --- | --- |
|  | 2007 | 2017 | Model 1^a^ | Model 2^b^ | Model 3^c^ |
| Overall | 11.7 (10.7-12.9) | 14.3 (13.6-14.9) | 1.24 (1.11 to 1.38)* | 1.24 (1.11 to 1.38)* | 1.19 (1.11-1.28)* |
| Sex | | | | | |
| Men | 13.9 (12.3-15.7) | 17.5 (16.4-18.6) | 1.30 (1.11 to 1.52)* | 1.29 (1.11 to 1.50)* | 1.24 (1.13-1.38)* |
| Women | 9.5 (8.8-10.3) | 11.0 (10.2-11.9) | 1.15 (1.01 to 1.30)* | 1.16 (1.02 to 1.31)* | 1.14 (1.01-1.27)* |
| Location | | | | | |
| Urban | 12.0 (10.3-14.0) | 14.0 (12.6-15.5) | 1.18 (0.97 to 1.44) | 1.18 (0.96 to 1.45) | 1.21 (1.00-1.46)* |
| Rural | 11.5 (8.9-14.6) | 14.6 (13.1-16.2) | 1.29 (0.97 to 1.71) | 1.29 (0.97 to 1.71) | 1.18 (0.97-1.44) |
| Age group | | | | | |
| 20-29 | 9.4 (7.5-11.8) | 13.2 (12.1-14.4) | 1.48 (1.13 to 1.94)* | 1.49 (1.13 to 1.96)* | 1.41 (1.15-1.74)* |
| 30-39 | 11.1 (9.7-12.6) | 14.5 (13.4-15.7) | 1.37 (1.15 to 1.63)* | 1.36 (1.14 to 1.62)* | 1.28 (1.08-1.52)* |
| 40-49 | 12.7 (11.6-13.9) | 15.8 (14.8-16.9) | 1.29 (1.14 to 1.47)* | 1.30 (1.14 to 1.48)* | 1.28 (1.13-1.44)* |
| 50-59 | 13.6 (12.7-14.5) | 14.7 (13.9-15.4) | 1.09 (0.99 to 1.20) | 1.10 (1.00 to 1.20) | 1.07 (0.97-1.18) |
| 60-69 | 14.0 (12.2-16.0) | 14.8 (13.8-16.0) | 1.07 (0.90 to 1.28) | 1.07 (0.90 to 1.28) | 0.98 (0.84-1.15) |
| ≥70 | 12.0 (9.2-15.4) | 11.5 (10.0-13.4) | 0.96 (0.69 to 1.34) | 0.93 (0.65 to 1.32) | 1.01 (0.69-1.48) |
| Ethnicity | | | | | |
| Han | 12.4 (11.8-13.1) | 14.4 (13.7-15.1) | 1.17 (1.09 to 1.27)* | 1.18 (1.09 to 1.27)* | 1.17 (1.10-1.25)* |
| Non-Han | 7.5 (6.1-9.1) | 11.5 (8.9-14.7) | 1.57 (1.10 to 2.24)* | 1.57 (1.07 to 2.31)* | 1.33 (0.90-1.95) |
| Income per year | | | | | |
| ≤30000 Yuan | 11.6 (10.2-13.1) | 14.2 (13.5-15.0) | 1.22 (1.06 to 1.40)* | 1.22 (1.07 to 1.40)* | 1.16 (1.05-1.27)* |
| >30000 Yuan | 11.4 (10.2-12.7) | 14.2 (13.4-15.0) | 1.30 (1.15 to 1.48)* | 1.33 (1.17 to 1.50)* | 1.29 (1.15-1.44)* |
| Education | | | | | |
| Less than college | 12.0 (10.5-13.6) | 14.4 (13.5-15.3) | 1.22 (1.06 to 1.40)* | 1.21 (1.05 to 1.39)* | 1.14 (1.04-1.25)* |
| College and above | 11.3 (10.2-12.6) | 14.0 (13.2-15.0) | 1.36 (1.20 to 1.56)* | 1.44 (1.26 to 1.64)* | 1.42 (1.26-1.62)* |
| Cigarette smoking | | | | | |
| Current non-smoker | 12.1 (11.2-13.0) | 14.4 (13.6-15.2) | 1.19 (1.07 to 1.31)* | 1.20 (1.08 to 1.32)* | 1.18 (1.09-1.29)* |
| Current smoker | 11.2 (9.6-12.9) | 14.4 (13.1-15.9) | 1.36 (1.11 to 1.66)* | 1.35 (1.11 to 1.65)* | 1.23 (1.08-1.39)* |
| Body mass index | | | | | |
| <23 | 13.6 (11.2-16.4) | 18.8 (17.7-20.0) | 1.33 (1.08-1.63)* | 1.44 (1.15-1.81)* | 1.40 (1.20-1.64)* |
| 23-<25 | 19.0 (17.0-21.2) | 23.8 (22.6-25.1) | 1.30 (1.11-1.52)* | 1.30 (1.11-1.51)* | 1.22 (1.07-1.40)* |
| ≥25 | 25.7 (24.4-27.1) | 28.2 (26.8-29.6) | 1.16 (1.05-1.28)* | 1.15 (1.05-1.26)* | 1.10 (1.02-1.19) |
| Waist circumference | | | | | |
| Men <90cm, women <80 cm | 11.0 (9.7-12.5) | 13.7 (12.9-14.6) | 1.24 (1.07 to 1.44)* | 1.27 (1.09 to 1.48)* | 1.24 (1.12-1.37)* |
| Men ≥90 cm, women ≥80 cm | 14.9 (14.1-15.8) | 16.6 (15.6-17.7) | 1.14 (1.04 to 1.25)* | 1.11 (1.01 to 1.21)* | 1.11 (1.02-1.22)* |

^a^ Model 1: Unadjusted.

^b^ Model 2: Adjusted for age and sex.

^c^ Model 3: Adjusted for age, sex, location, ethnicity, income level, education level, and smoking status, BMI, and waist circumference.

^*^ indicates a *P*-value less than 0.05.

**Supplementary Table 7. Changes in age- and sex-standardized prevalence of grade 1 hypertension between 2007 and 2017 in adults in China by subgroups.**

|  | Prevalence (95% CI) | | Odds ratio (95% CI) for change from 2007 to 2017 | | |
| --- | --- | --- | --- | --- | --- |
|  | 2007 | 2017 | Model 1^a^ | Model 2^b^ | Model 3^c^ |
| Overall | 18.2 (15.9-20.7) | 21.4 (20.2-22.6) | 1.17 (0.97 to 1.42) | 1.23 (1.01 to 1.49)* | 1.08 (0.93-1.26) |
| Sex | | | | | |
| Men | 20.3 (17.1-23.9) | 25.9 (24.4-27.4) | 1.30 (1.03 to 1.65)* | 1.37 (1.08 to 1.74)* | 1.16 (0.97-1.38) |
| Women | 16.1 (14.6-17.7) | 16.8 (15.5-18.2) | 1.02 (0.85 to 1.22) | 1.04 (0.87 to 1.24) | 1.00 (0.85-1.17) |
| Location | | | | | |
| Urban | 19.2 (16.9-21.7) | 20.3 (18.8-21.9) | 0.98 (0.81 to 1.18) | 1.07 (0.87 to 1.31) | 1.01 (0.83-1.23) |
| Rural | 17.4 (13.22-22.6) | 22.5 (20.8-24.3) | 1.39 (0.98 to 1.98) | 1.39 (0.96 to 2.01) | 1.13 (0.90-1.42) |
| Age group | | | | | |
| 20-29 | 6.0 (5.1-7.1) | 8.7 (7.8-9.7) | 1.51 (1.23 to 1.85)* | 1.57 (1.26 to 1.96)* | 1.46 (1.17-1.83)* |
| 30-39 | 10.2 (8.1-12.8) | 13.8 (12.4-15.3) | 1.43 (1.08 to 1.89)* | 1.44 (1.08 to 1.93)* | 1.18 (0.89-1.55) |
| 40-49 | 19.2 (17.7-20.9) | 23.1 (21.1-25.3) | 1.27 (1.09 to 1.48)* | 1.28 (1.09 to 1.50)* | 1.14 (0.99-1.32) |
| 50-59 | 26.9 (22.2-32.2) | 31.4 (29.7-33.3) | 1.25 (0.95 to 1.63) | 1.23 (0.94 to 1.61) | 1.09 (0.90-1.33) |
| 60-69 | 35.7 (32.2-39.5) | 36.9 (34.7-39.1) | 1.05 (0.87 to 1.27) | 1.05 (0.88 to 1.27) | 0.94 (0.80-1.11) |
| ≥70 | 39.3 (32.6-46.5) | 40.8 (38.4-43.2) | 1.06 (0.78 to 1.45) | 1.08 (0.80 to 1.46) | 0.96 (0.71-1.30) |
| Ethnicity | | | | | |
| Han | 19.4 (17.9-21.1) | 21.6 (20.4-22.9) | 1.08 (0.93 to 1.26) | 1.14 (0.99 to 1.31) | 1.07 (0.92-1.24) |
| Non-Han | 10.0 (7.0-14.1) | 16.1 (13.8-18.7) | 1.72 (1.19 to 2.49)* | 1.78 (1.12 to 2.82)* | 1.20 (0.91-1.57) |
| Income per year | | | | | |
| ≤30000 Yuan | 18.0 (15.7-20.7) | 21.6 (20.2-23.2) | 1.32 (1.07 to 1.65)* | 1.22 (0.99 to 1.51) | 1.06 (0.92-1.21) |
| >30000 Yuan | 18.7 (15.6-22.2) | 21.0 (19.8-22.2) | 1.09 (0.84 to 1.40) | 1.20 (0.92 to 1.56) | 1.13 (0.87-1.46) |
| Education | | | | | |
| Less than college | 18.4 (15.7-21.3) | 22.5 (21.3-23.8) | 1.35 (1.09 to 1.66)* | 1.27 (1.02 to 1.58)* | 1.08 (0.91-1.28) |
| College and above | 17.7 (16.1-19.5) | 18.5 (17.0-20.1) | 0.99 (0.79 to 1.23) | 1.19 (1.00 to 1.41) | 1.12 (0.93-1.35) |
| Cigarette smoking | | | | | |
| Current non-smoker | 19.1 (17.1-21.2) | 21.7 (20.4-23.0) | 1.07 (0.90 to 1.28) | 1.13 (0.96 to 1.33) | 1.06 (0.91-1.23) |
| Current smoker | 14.9 (11.8-18.6) | 21.8 (20.1-23.7) | 1.46 (1.12 to 1.91)* | 1.49 (1.12 to 2.00)* | 1.14 (0.95-1.36) |
| Body mass index | | | | | |
| <23 | 11.3 (9.0-14.1) | 14.3 (13.0-15.8) | 1.14 (0.87-1.48) | 1.28 (0.95-1.71) | 1.19 (0.95-1.49) |
| 23-<25 | 17.9 (16.0-20.0) | 20.4 (18.8-22.0) | 1.16 (0.96-1.41) | 1.17 (0.96-1.42) | 1.08 (0.88-1.32) |
| ≥25 | 27.4 (25.4-29.5) | 28.7 (27.6-29.8) | 1.08 (0.94-1.23) | 1.09 (0.96-1.24) | 1.03 (0.89-1.19) |
| Waist circumference | | | | | |
| Men <90cm, women <80 cm | 14.3 (12.0-17.0) | 17.3 (16.1-18.5) | 1.16 (0.93 to 1.44) | 1.25 (0.99 to 1.58) | 1.19 (0.99-1.43) |
| Men ≥90 cm, women ≥80 cm | 28.2 (26.2-30.2) | 28.3 (26.3-30.3) | 0.95 (0.82 to 1.11) | 0.98 (0.85 to 1.14) | 0.96 (0.82-1.12) |

^a^ Model 1: Unadjusted.

^b^ Model 2: Adjusted for age and sex.

^c^ Model 3: Adjusted for age, sex, location, ethnicity, income level, education level, and smoking status, BMI, and waist circumference.

^*^ indicates a *P*-value less than 0.05.

**Supplementary Table 8. Changes in age- and sex-standardized prevalence of grade 2 hypertension between 2007 and 2017 in adults in China by subgroups.**

|  | Prevalence (95% CI) | | Odds ratio (95% CI) for change from 2007 to 2017 | | |
| --- | --- | --- | --- | --- | --- |
|  | 2007 | 2017 | Model 1^a^ | Model 2^b^ | Model 3^c^ |
| Overall | 7.4 (6.5-8.4) | 9.4 (7.7-11.5) | 1.23 (0.94 to 1.61) | 1.27 (0.97 to 1.66) | 1.18 (0.91-1.53) |
| Sex | | | | | |
| Men | 8.5 (7.3-10.0) | 11.2 (8.9-14.0) | 1.28 (0.94 to 1.74) | 1.33 (0.98 to 1.81) | 1.21 (0.90-1.63) |
| Women | 6.3 (5.6-7.0) | 7.6 (6.2-9.2) | 1.16 (0.89 to 1.52) | 1.18 (0.91 to 1.52) | 1.14 (0.89-1.45) |
| Location | | | | | |
| Urban | 7.2 (5.6-9.3) | 7.3 (6.3-8.4) | 0.91 (0.65 to 1.27) | 0.98 (0.70 to 1.38) | 1.01 (0.73-1.41) |
| Rural | 7.6 (5.6-10.2) | 11.6 (8.6-15.4) | 1.59 (1.04 to 2.43)* | 1.56 (0.99 to 2.47) | 1.31 (0.86-1.98) |
| Age group | | | | | |
| 20-29 | 1.3 (0.9-2.0) | 2.8 (1.1-7.4) | 2.21 (0.75 to 6.52) | 2.33 (0.82 to 6.63) | 2.35 (0.76-7.33) |
| 30-39 | 3.7 (3.2-4.2) | 5.3 (3.2-8.7) | 1.50 (0.87 to 2.59) | 1.53 (0.89 to 2.64) | 1.44 (0.73-2.85) |
| 40-49 | 8.1 (6.7-9.7) | 8.9 (7.7-10.2) | 1.11 (0.86 to 1.44) | 1.12 (0.86 to 1.45) | 1.05 (0.82-1.35) |
| 50-59 | 12.4 (10.4-14.8) | 13.6 (12.0-15.5) | 1.11 (0.86 to 1.43) | 1.10 (0.86 to 1.41) | 0.99 (0.80-1.24) |
| 60-69 | 15.6 (13.9-17.5) | 19.2 (16.8-21.8) | 1.29 (1.04 to 1.59)* | 1.29 (1.05 to 1.60)* | 1.16 (0.96-1.41) |
| ≥70 | 16.2 (12.6-20.5) | 23.8 (20.2-27.9) | 1.62 (1.13 to 2.32)* | 1.57 (1.10 to 2.25)* | 1.44 (1.01-2.07)* |
| Ethnicity | | | | | |
| Han | 7.8 (6.8-8.9) | 9.5 (7.7-11.7) | 1.17 (0.88 to 1.55) | 1.22 (0.92 to 1.61) | 1.18 (0.90-1.55) |
| Non-Han | 4.8 (3.5-6.6) | 6.5 (5.6-7.5) | 1.37 (1.00 to 1.88) | 1.37 (0.93 to 2.01) | 0.92 (0.76-1.11) |
| Income per year | | | | | |
| ≤30000 Yuan | 7.7 (6.8-8.8) | 10.4 (8.2-13.1) | 1.47 (1.12 to 1.92)* | 1.33 (1.01 to 1.75)* | 1.14 (0.88-1.47) |
| >30000 Yuan | 6.1 (4.3-8.7) | 8.6 (7.0-10.4) | 1.30 (0.84 to 2.02) | 1.44 (0.94 to 2.22) | 1.37 (0.92-2.05) |
| Education | | | | | |
| Less than college | 7.6 (6.5-8.9) | 10.5 (8.1-13.5) | 1.44 (1.11 to 1.87)* | 1.35 (1.02 to 1.79)* | 1.19 (0.91-1.56) |
| College and above | 6.2 (4.8-7.9) | 7.0 (5.8-8.4) | 0.96 (0.71 to 1.32) | 1.16 (0.86 to 1.57) | 1.18 (0.83-1.67) |
| Cigarette smoking | | | | | |
| Current non-smoker | 7.7 (6.8-8.7) | 9.2 (7.8-10.9) | 1.15 (0.90 to 1.48) | 1.20 (0.95 to 1.52) | 1.17 (0.93-1.47) |
| Current smoker | 7.7 (5.5-10.6) | 9.8 (7.3-13.0) | 1.43 (1.00 to 2.05)* | 1.45 (1.00 to 2.12) | 1.20 (0.82-1.76) |
| Body mass index | | | | | |
| <23 | 3.9 (3.5-4.4) | 5.9 (4.5-7.7) | 1.37 (0.92-2.04) | 1.51 (1.04-2.18)* | 1.41 (0.98-2.03) |
| 23-<25 | 6.1 (5.4-6.8) | 8.5 (6.8-10.5) | 1.37 (1.06-1.77)* | 1.37 (1.06-1.77)* | 1.34 (1.04-1.72)* |
| ≥25 | 12.2 (10.7-14.0) | 12.9 (10.5-15.8) | 1.03 (0.80-1.32) | 1.04 (0.81-1.32) | 1.05 (0.83-1.34) |
| Waist circumference | | | | | |
| Men <90cm, women <80 cm | 5.1 (4.6-5.7) | 7.3 (5.6-9.4) | 1.32 (0.94 to 1.86) | 1.41 (1.00 to 1.99)* | 1.41 (1.01-1.96)* |
| Men ≥90 cm, women ≥80 cm | 12.9 (11.2-14.8) | 12.8 (10.6-15.3) | 0.94 (0.73 to 1.21) | 0.97 (0.76 to 1.24) | 0.99 (0.78-1.26) |

^a^ Model 1: Unadjusted.

^b^ Model 2: Adjusted for age and sex.

^c^ Model 3: Adjusted for age, sex, location, ethnicity, income level, education level, and smoking status, BMI, and waist circumference.

^*^ indicates a *P*-value less than 0.05.


**Supplementary Table 9. Unadjusted and ages-sex-adjusted changes in mean body mass index and waist circumference over 10 years among adults in mainland China.** Values are means (95% confidence intervals)

|  | Body mass index | | | | Waist circumference | | | |
| --- | --- | --- | --- | --- | --- | --- | --- | --- |
|  | Unadjusted difference | p value | Adjusted difference | p value | Unadjusted difference | p value | Adjusted difference | p value |
| Overall | 0.4 (0.01 to 0.7) | 0.04 | 0.4 (0.1 to 0.8) | 0.02 | 2.7 (1.4 to 4.1) | 0.0002 | 2.9 (1.5 to 4.3) | 0.0001 |
| Sex | | | | | | | | |
| Men | 0.7 (0.1 to 1.2) | 0.03 | 0.7 (0.1 to 1.3) | 0.02 | 3.4 (1.3 to 5.6) | 0.003 | 3.6 (1.4 to 5.7) | 0.002 |
| Women | 0.1 (-0.2 to 0.3) | 0.52 | 0.1 (-0.1 to 0.4) | 0.2 | 2.0 (0.9 to 3.0) | 0.0005 | 2.2 (1.3 to 3.2) | <0.0001 |
| Age group | | | | | | | | |
| 20-29 | 0.2 (-0.1 to 0.5) | 0.2 | 0.3 (0.01 to 0.7) | 0.048 | 2.3 (0.9 to 3.7) | 0.002 | 2.7 (1.3 to 4.0) | 0.0002 |
| 30-39 | 0.6 (0.4 to 0.7) | <0.0001 | 0.6 (0.4 to 0.8) | <0.0001 | 3.6 (2.8 to 4.4) | <0.0001 | 3.6 (2.7 to 4.4) | <0.0001 |
| 40-49 | 0.5 (0.2 to 0.8) | 0.002 | 0.5 (0.2 to 0.8) | 0.002 | 2.9 (1.6 to 4.2) | <0.0001 | 2.9 (1.6 to 4.2) | 0.0001 |
| 50-59 | 0.6 (0.01 to 1.2) | 0.046 | 0.6 (0.02 to 1.2) | 0.04 | 3.6 (1.7 to 5.4) | 0.0004 | 3.5 (1.6 to 5.4) | 0.0005 |
| 60-69 | 0.7 (-0.1 to 1.5) | 0.1 | 0.7 (-0.1 to 1.5) | 0.1 | 3.5 (0.8 to 6.1) | 0.01 | 3.5 (0.9 to 6.2) | 0.01 |
| ≥70 | 0.3 (-0.3 to 0.9) | 0.36 | 0.4 (-0.2 to 1.0) | 0.24 | 2.0 (0.4 to 3.6) | 0.02 | 2.1 (0.7 to 3.6) | 0.005 |
| Location | | | | | | | | |
| Urban | 0.0 (-0.6 to 0.6) | 0.99 | 0.1 (-0.4 to 0.7) | 0.69 | 1.3 (-0.5 to 3.1) | 0.14 | 1.7 (0.1 to 3.4) | 0.04 |
| Rural | 0.7 (-0.1 to 1.5) | 0.09 | 0.7 (-0.1 to 1.5) | 0.1 | 4.1 (1.0 to 7.2) | 0.01 | 4.0 (0.9 to 7.1) | 0.01 |

**Supplementary Table 10. Unadjusted and ages-sex-adjusted changes in mean blood pressure over 10 years among adults in mainland China.** Values are means (95% confidence intervals)

|  | Systolic blood pressure | | | | Diastolic blood pressure | | | |
| --- | --- | --- | --- | --- | --- | --- | --- | --- |
|  | Unadjusted difference | p value | Adjusted difference | p value | Unadjusted difference | p value | Adjusted difference | p value |
| Overall | 4.9 (2.8 to 7.0) | <0.0001 | 5.4 (3.5 to 7.4) | <0.0001 | 1.0 (-0.7 to 2.7) | 0.23 | 1.2 (-0.5 to 2.8) | 0.17 |
| Sex |  |  |  |  |  |  |  |  |
| Men | 6.6 (4.2 to 9.0) | <0.0001 | 7.1 (4.8 to 9.5) | <0.0001 | 1.6 (-0.5 to 3.8) | 0.13 | 1.8 (-0.3 to 3.9) | 0.09 |
| Women | 3.1 (1.0 to 5.2) | 0.004 | 3.7 (2.0 to 5.5) | 0.0001 | 0.3 (-1.0 to 1.7) | 0.61 | 0.5 (-0.8 to 1.8) | 0.43 |
| Age group | | | | | | | | |
| 20-29 | 5.7 (4.6 to 6.9) | <0.0001 | 5.7 (4.6 to 6.9) | <0.0001 | 1.5 (-0.2 to 3.3) | 0.08 | 1.7 (-0.04 to 3.4) | 0.06 |
| 30-39 | 5.5 (4.1 to 6.9) | <0.0001 | 5.5 (4.1 to 6.9) | <0.0001 | 1.6 (-0.1 to 3.4) | 0.06 | 1.7 (-0.1 to 3.4) | 0.06 |
| 40-49 | 5.3 (3.4 to 7.2) | <0.0001 | 5.3 (3.4 to 7.2) | <0.0001 | 1.2 (-0.4 to 2.8) | 0.13 | 1.2 (-0.4 to 2.8) | 0.13 |
| 50-59 | 5.1 (1.9 to 8.3) | 0.003 | 4.8 (1.7 to 8.0) | 0.004 | 1.0 (-1.2 to 3.2) | 0.39 | 0.9 (-1.3 to 3.1) | 0.4 |
| 60-69 | 4.7 (1.5 to 7.9) | 0.005 | 4.7 (1.6 to 7.9) | 0.004 | 0.9 (-1.1 to 2.8) | 0.38 | 0.9 (-1.1 to 2.8) | 0.38 |
| ≥70 | 6.2 (2.4 to 10.0) | 0.002 | 5.8 (1.8 to 9.7) | 0.005 | 0.7 (-1.5 to 3.0) | 0.51 | 1.2 (-1.0 to 3.4) | 0.29 |
| Location | | | | | | | | |
| Urban | 1.9 (-0.8 to 4.5) | 0.17 | 2.9 (0.2 to 5.6) | 0.03 | -0.6 (-2.5 to 1.3) | 0.51 | -0.3 (-2.2 to 1.5) | 0.74 |
| Rural | 8.0 (3.6 to 12.4) | 0.0006 | 7.9 (3.5 to 12.2) | 0.0007 | 2.6 (-0.7 to 6.0) | 0.12 | 2.6 (-0.8 to 5.9) | 0.13 |
